# The Chinese customers and service staff interactive affective system (CCSIAS): introduction to a multimodal stimulus dataset

**DOI:** 10.3389/fpsyg.2024.1302253

**Published:** 2024-05-03

**Authors:** Ping Liu, Yi Zhang, Ziyue Xiong, Ying Gao

**Affiliations:** School of Business, Sichuan University, Chengdu, China

**Keywords:** customers, service staff, interactive affective system, natural workplace, multimodal dataset

## Abstract

To research the emotional interaction between customers and service staff, single-modal stimuli are being used to activate subjects’ emotions while multimodal emotion stimuli with better efficiency are often neglected. This study aims to construct a multimodal emotion stimuli database (CCSIAS) with video records of real work status of 29 service staff and audio clips of interactions between customers and service staff by setting up wide-angle cameras and searching in company’s Ocean Engine for 15 consecutive days. First, we developed a tool to assess the emotional statuses of customers and service staff in Study 1. Second, 40 Masters and PhD students were invited to assess the audio and video data to evaluate the emotional states of customers and service staff in Study 2, using the tools developed in Study 1. Third, 118 participants were recruited to test the results from Study 2 to ensure the stability of the derived data. The results showed that 139 sets of stable emotional audio & video data were constructed (26 sets were high, 59 sets were medium and 54 sets were low). The amount of emotional information is important for the effective activation of participants’ emotional states, and the degree of emotional activation of video data is significantly higher than that of the audio data. Overall, it was shown that the study of emotional interaction phenomena requires a multimodal dataset. The CCSIAS (https://osf.io/muc86/) can extend the depth and breadth of emotional interaction research and can be applied to different emotional states between customers and service staff activation in the fields of organizational behavior and psychology.

## 1 Introduction

In complex customer service environments, the subtle dynamics of emotional interactions play a crucial role in shaping customer satisfaction and service quality. The process of emotional interaction originates from the encoding and interpretation of emotional information by the human brain. This unconscious process is a multimodal problem involving tones, movements, and more. However, despite increasing interest in this field, the currently available multimodal emotion databases are very limited, especially in the context of Chinese culture.

In response to this critical gap, this study aims to design and construct a pioneering multimodal database named China Customer Service Interaction Emotional System (CCSIAS), and evaluate the emotional states of customers and service personnel during the service provision process. It contains comprehensive video and audio data collected from real work environments over a long period of time, meticulously records emotional communication between customers and service staff.

This study will also decipher the complex emotional narratives contained in this rich dataset, aiming to make a significant contribution to understanding and improving emotional intelligence in customer service environments, ultimately cultivating more empathetic and effective service interactions.

## 2 Theoretical background

**Figure 1:**
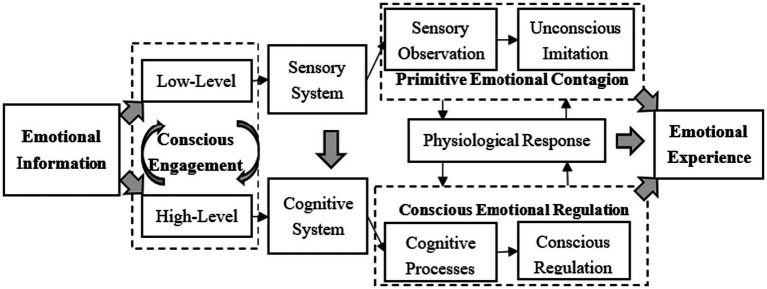
The evoking mechanisms of emotional experience.

The emotional interaction between customers and service staff refers to a process of emotional convergence, that is, the tendency of one person to imitate the facial expressions, body actions, and communication voices of the other when influenced by the other’s emotional state (Hatfield et al., 1993; Belkin, 2009; Zhang et al., 2016; Fang et al., 2019; Liu et al., 2022). Emotional interactions are central to service quality and performance, and play an invaluable role in service delivery (Liao et al., 2009; Liu et al., 2019). As such, studies of emotional interactions have received considerable scholars’ attention in recent years (Rueff-Lopes et al., 2015; Grandey and Melloy, 2017; Liu et al., 2022; VanKleef and Cote, 2022). Primitive emotional contagion (see Figure 1) means that people pick up verbal and non-verbal cues from others through the sensory system (Hatfield et al., 1993), and then automatically and unconsciously imitate the emotional states of others (Du et al., 2011), thus achieving emotional assimilation (Hennig-Thurau et al., 2006; Zhang et al., 2016). With the high-interaction and high-participation nature of service delivery (Rueff-Lopes et al., 2015; Liu et al., 2022), service-staff are often asked to manage and express their emotions in line with organizational goals. This phenomenon has been characterized as emotional labor (Hochschild, 1979; Ni and Li, 2021), which requires the engagement of higher cognitive systems such as the brain (Zhang and Lu, 2013). Therefore, although the sensory system provides direct access to emotional information during the service process, service staff still need to utilize the cognitive system to regulate their emotions when delivering service to customers. In summary, the emotional interaction in this study is characterized as an experience that includes two components: primitive emotional contagion based on unconscious imitation (Hatfield et al., 1993) and conscious emotional regulation brought about by the engagement of the cognitive system (Falkenberg et al., 2008; Du et al., 2011; Zhang and Lu, 2013).

Emotional information is the basis from which emotional experiences emerge that allows emotional interactions to occur. It can be categorized into two types: low-level emotional information and high-level emotional information (Hoffman, 2002; Hennig-Thurau et al., 2006; Du et al., 2011), which can be distinguished by the existence of conscious engagement (Zhang and Lu, 2013). Low-level emotional information, which refers mainly to sensory emotional information (e.g., facial expressions, body actions, scents, etc.), is presented without acquired learning, and can be perceived directly through the sensory system with cross-species uniformity (Kiss and Eimer, 2008). More recently, it has been confirmed that it is a prerequisite for emotional interaction or emotional contagion (Du et al., 2011; Aviezer et al., 2012; Zhang and Lu, 2015). Meanwhile, high-level emotional information (e.g., text, music, films, etc.) can be encoded and interpreted by the cognitive system, such as the human brain (Falkenberg et al., 2008). As high-level emotional information requires the involvement of consciousness, it is used by researchers in the fields of organizational behavior and psychology to construct and establish emotion-specific stimulus materials to activate and awaken specific emotions (Bai et al., 2005; Zhao et al., 2017; Liu et al., 2020; Rosalie et al., 2020; Yuan et al., 2021; Zhou et al., 2021; David et al., 2022).

Unconscious activation of emotions is essential for studying the phenomena of emotional interaction, which is a multimodal question. Multimodality, is a neologism: when multi is affixed to modality, the composite word means a combination of several compositions. A modality is essentially a system of social symbols that can be perceived by a person, and a characteristic method of presenting and delivering emotions to others (Lackovi, 2018; Perveen et al., 2020). In terms of data storage structure, if a piece of emotional information can be encoded and stored in a single methodology and can be interpreted by a single sensory system, then this emotional data can be identified as single-modal. However, if the emotional information is encoded and stored in two or more methodologies and must be interpreted by more than one sensory system, the emotional data is multimodal (Kress, 2010; Lahat et al., 2015; Wang et al., 2021; Liu et al., 2022). To date, a series of studies in organizational behavior, marketing and tourism research have confirmed that reliance on single-modal data can effectively activate a participant’s cognitive or sensory system to achieve different emotional arousal. For example, scholars have also asked participants to acquire emotional information through the visual or auditory sensory systems, and then process single-modality emotions such as pictures (Lang et al., 1997; Bai et al., 2005; Elizabeth et al., 2017), music (Rosalie et al., 2020), and text (Du et al., 2012; Beth et al., 2013; Hans et al., 2017; Xu et al., 2022), etc., with the help of cognitive system to active specific emotions. However, the emotional interaction between customers and service staff is usually accompanied by three kinds of emotional communication of information, namely: facial expressions, body actions and voice. Due to the presence of emotional labor, service delivery was essentially a process in which the service staff’s cognitive system process his or her own facial expression and voice, which were then captured and processed by the customers’ sensory system, resulting in a positive emotional experience (Foroni and Semin, 2009; Rueff-Lopes et al., 2015; Liu et al., 2022). Then, single-modality emotional stimulation experiments such as pictures and music are not only ineffective in activate participant’s emotional experience, but also difficult to convey the true emotional state of service staff, leading to biased results of emotional activation (Liu et al., 2022). In addition, human emotions are rich and complex (Perveen et al., 2020), a positive sentence may contain both irony and bitterness. On the other hand, the emotions of service staff during the delivery process are usually expressed as a combination of discrete emotions. Therefore, it is necessary to construct a multimodal dataset to effectively study the emotional interactions between customers and service staff based on conveying the real emotions of service staff.

In the aspect of service practice, online service represented by voice-to-voice has become an important method of delivering services to customers in the modern service industry (Rueff-Lopes et al., 2015; Liu et al., 2022). Unlike traditional off-line service which uses face-to-face interactions, the voice-to-voice communication between customers and service staff has become an important part of the service delivery process (Rueff-Lopes et al., 2015). In this situation, customers are more likely to introduce negative emotions, which can seriously affect the emotional state of service staff. However, previous research has not paid enough attention to the issue of customer-customer service emotional interactions. If that any researchers are cognizant of it, they tend to choose stimulus materials for their own practical needs (Rueff-Lopes et al., 2015; Sun et al., 2018) and are susceptible to constraint such as inadequate standardization of stimulus materials. Specifically, there are five flaws in the current emotional databases. First, the other-race effects are overlooked (Malpass and Kravitz, 1969) when current databases are applied to activate the emotions of online service staff. While cultural, ethnic, and national differences cannot be ignored. Under the influence of traditional Chinese culture, Chinese people express their emotions in a more subtle and ambiguous way, but there are very few emotion databases based on Chinese people. Second, existing databases are mostly based on the representations of professional actors but rarely on real-life situations, which often fails to capture the details of real-life scenarios that could most activate emotions. Third, discrete emotions (e.g., fear, disgust, sadness, curiosity, etc.) are difficult to distinguish in reality, so existing databases have limitations when being used for emotional interactions. Fourth, multimodal databases based on both visual and auditory senses are rare. Some of the materials in such databases are mostly generated from films or popular music, and thus participants’ previous encounter of the materials may have a negative impact on the outcome of emotional activation. Fifth, the existing databases still lack clear labels for the degree of emotional interaction between customers and service staff.

In response to these problems, this article designed the following process to collect data and build a database: First, researchers set up wide-angle cameras and an Ocean Engine to record the 29-work status of service staff using video (visual and auditory) and audio (auditory) modalities for 15 consecutive days. Then, three sub-studies were designed to construct CCSIAS. Finally, volunteers were recruited into the study to test the stability and validity of the CCSIAS.

## 3 Data acquisition methods for CCSISA

### 3.1 Methods

Twenty nine telemarketing service staff (both pre-sales and post-sales positions) from the Call Center of a large decoration company were invited to participate in our study. Among them, 6 (20.69%) and 23 (79.31%) of the service staff were identified as female and male respectively, with an age range of 19 ~ 33 years (*M* = 27.556, SD = 1.144). More than four-fifths (86.21%) of the participants had a university degree, and the average length of service was 6.08 (SD = 0.972) years.

All participants voluntarily signed an informed consent that: (a) they understood the purpose of the study and were willing to participate; (b) the method of data collection, based on wide-angle cameras, was undertaken with their permission and consent; (c) they gave the research team permission to record their physical and psychological data; (d) all facial images would be stored in accordance with legal rules. This study was registered with the Ethics Committee of Sichuan University (No. KS2022984).

### 3.2 Materials

The data was collected by wide-angle cameras (Types: AIGO DSJ-T5) and the company’s Ocean Engine cloud. The data was collected continuously for 2-weeks, from 9:00 am to 18:00 pm every day from 15 to 30 March, 2021.

**Figure 2:**
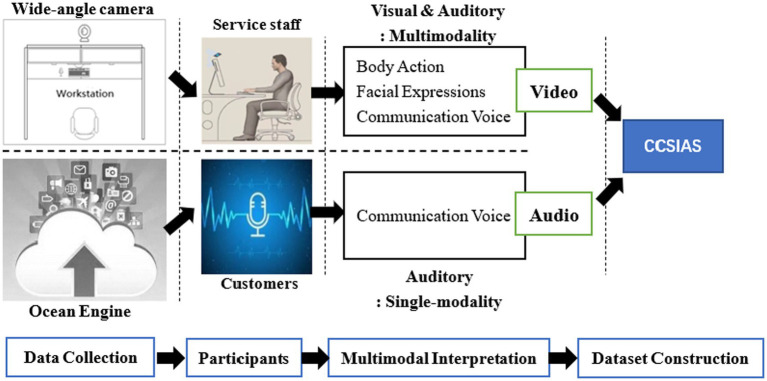
Data acquisition schematic.

There are three reasons for this choice: (1) this company is a leading enterprise in China’s home decoration industry, whose business covers all major cities, such as Beijing, Shanghai, Guangzhou, and Wuhan etc. (2) March 15 is the beginning of the “3·15 Quality Home Decoration Festival” in the industry. There is abundant telemarketing business in this period of time, and the frequency of service delivery is high and the length of service is sufficiently long, making it easier for the researchers to capture the emotional changes of service staff and providing wide-range emotional data for CCSIAS. (3) The wide-angle cameras were able to capture the real working video emotions of service staff, while the Ocean Engine can download the audio emotions of customers, which helps to study the phenomenon of emotional interaction based on real emotional data (see Figure 2).

In order to minimize the potential resistance and discomfort of service staff, prior to data collection, the supervisor and manager explained the meaning of this study to each member of the service staff. During the collection, the research team’s manager told each participant that the data collected in this study would not be submitted to third parties and would not cause any negative influence on their career. After collection, the research team gave a generous reward to service staff who successfully finished this study.

### 3.3 Results

During the collection process, three service staff showed obvious hostile behavior (e.g., deliberately blocking or turning off the camera, making it impossible to capture video data), and one individual resigned for personal reasons. In order not to cast negative impact on the four-service staff’s work, we removed the cameras from their work stations. In total, this study collected 25 service staff’s emotional video data, with 9215GB of original recordings, and the efficiency rate reached 89.99% (=25÷29). In addition, in order to avoid evaluation errors caused by the video format and playback quality, we transcoded the video by using Adobe Premier Pro 2021, adjusting the resolution of the video to 960 × 540, in which the frame rate of the image was 30.0 fps and the sound sampling frequency was 48.0 kHz. All data was saved in MP4 format. Audio data was downloaded from the company’s cloud-based Ocean Engine. A total of 16,115 audio phone calls, corresponding to the video, were made by the 25 service staff in half a month.

## 4 Study1: a free association test of emotional interactions between customers and service staff

Study 1 aims to construct a set of tools to evaluate the real emotional state of customers and service staff in online service delivery situations. It can provide picture and text-based scales for Study 2 and 3 to accurately evaluate the real emotional states between customers and service staff and the level of emotional interaction.

### 4.1 Methods

Twenty PhD students from Sichuan University and Yunnan University studying in the field of organizational psychology and computerized emotion recognition were recruited to participate in our study. Among them, 10 (50%) and 10 (50%) were identified as female and male respectively, aged 23 ~ 34 years ( M=26.401 , SD=0.654 ).

Specifically, participants were required to be (1) non-color blind and right-handed, (2) have normal or corrected-to-normal vision and hearing, (3) must have at least 2 years of research experience in emotion-related research filed and have published at least 2 academic articles in high-impact journals.

### 4.2 Procedure

**Figure 3:**
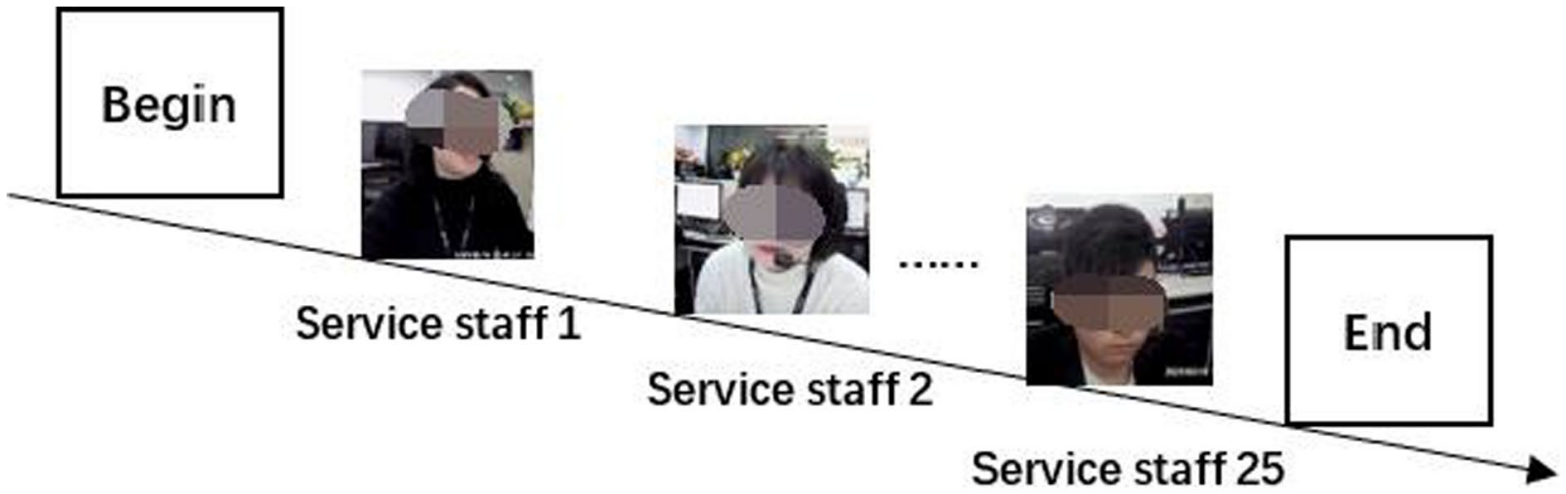
Flowchart of video playback.

Study 1 consisted of two consecutive tasks. Task 1. Participants were asked to observe the stats of each service staff at the workplace over 15 days, and then to describe their emotional condition when the staff was providing service to customers. In particular, the text should not use highly abstract and generalized words (e.g., “in normal states,” “sb’s heart leaps,” etc.). The video was played by using QQ Player. If the service staff exhibited significant emotional changes before and after the phone calls, the participants were asked to record the time of the service. At the same time, the emotional states of the service staff were labeled by the participants (valence: negative-neutral-positive; arousal: low-medium-high). Finally, the study required participants to make a quick judgment of the emotional state of the service staff, and to fill in only one set of the most compatible labels for each service delivery process. In addition, due to the presence of the fatigue effect, the study required the participants to take a break after a certain number of labels, with the duration of the break time determined autonomously (see Figure 3).

Based on the statistical analysis of the textual descriptions (Task 1), a text-based scale of service staff was constructed. Next, for each service staff, 5 ~ 10 pictures were captured from the video that most accurately reflected the emotional state. Last, 20 PhD students were asked to reverse vote on the pictures; the 5 ~ 7 pictures with the highest votes were eliminated, and the remaining pictures were combined using the SAM scale (Bradley and Lang, 1994) to create a picture-based scale.

### 4.3 Results

Study 1 cumulatively generated about 168,000 textual records of the emotional state of service staff when delivering service to customers, with invalid text eliminated. From the final texts, the most frequently appearing texts were selected to demonstrate the emotional state of service staff. For example, frowning, rolling eyes, deflating mouth, smiling, pouting, etc. were classified as Facial Expressions (FE). The text low, cursing, speaking faster, etc. were classified as Communication Voice (CV). Dropping the pen, shaking the head, resting the face with hands, etc. were classified as Body Actions (BA).

Thus, based on the frequency of texts and the emotional state of service staff, while referring to Bradley and Lang (1994) and Liu et al. (2022)’s 2 × 3 classification of emotional dimensions, we construct a text-based scale (see Table 1).

**Table 1:** Text-based scale for service staff’s emotional states (an example).

Emotional interval	Emotional state description
Negative and Low∈[1,4]	*Valence*: Always frowning, rolling their eyes, yawning……(FE); Embarrassing smile, speaking faster, escalating tone……(CV); Dropping the pen, leaning back in the chair, propping his head on his hand, and stretched......(BA).
	*Arousal*: the emotional status is very low, and activity is the lowest.
Neutral and Medium∈(4,6]	*Valence*: Calm facial expression……(FE); Medium speaking and appropriate speech intonation……(CV); Sitting upright and unchanged……(BA).
	*Arousal*: the emotional status is a quiet condition, and activity is normal.
Positive and High∈(6,9]	*Valence:* Answers questions with a smile, upturned mouth ...... (FE); the tone is soothing with, communication with a smile, sounding light-hearted……. (CV); Body sways, keeps swaying ...... (BA).
	*Arousal*: the emotional status is very excited, and activity is at the peak.

**Figure 4:**
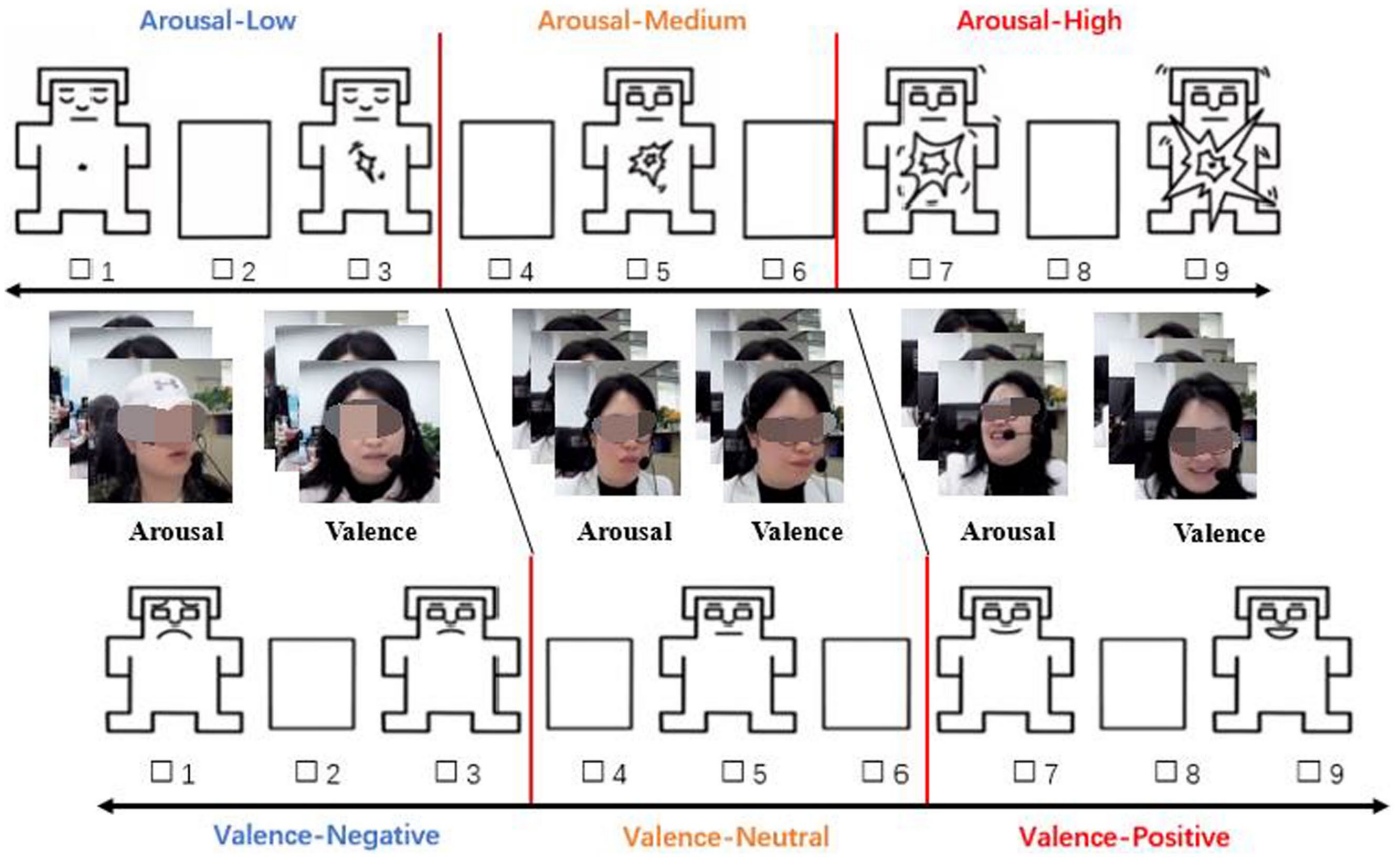
SAM picture-scale for service-staff’s emotional states.

Referring to Liu et al. (2022) and SAM (Bradley and Lang, 1994), we constructed picture-based scales separately for each service staff in this study by combining two dimensions of emotional valence and arousal (see Figure 4).

To test the applicability and validity of the assessment tool, 50% of the 20 subjects were randomly recruited for retesting. Participants were asked to re-fill in the emotion labels for each customer service emotional state, prompted by a picture and text-based scale. The data showed a high degree of correlation between the measures of the two experiments ( ICC=0.878,P<0.000 ) and a decrease in the sample variance between the two experiments from 1.934 to 0.528. Therefore, there is evidence that the Picture and Text Type Scale can be used to assist in assessing the true emotional state of customer service.

## 5 Study 2: construction of the database of Chinese customers and service staff interactive system

Using the emotional scales developed in Study 1, 40 participants were asked to listen to the audio and to watch the video of the service delivery process. The participants were then asked to evaluate customers’ and service staff’s emotional state in Study 2 to label the emotional scores in two modalities, thus providing the label pool for the data in the CCSIAS.

### 5.1 Methods

Twenty Masters students whose research areas were organizational psychology and computerized emotion recognition were openly recruited via posters and WeChat. In addition, the 20 PhD students from Study 1 were invited again to engage in the experiment. The main reason for re-inviting the PhD students is that they had already been involved in constructing the text and picture-based scales and had generated a comparatively consistent impression of the emotional state of customers and service staff. Therefore, they were able to reasonably judge the real emotions of the service staff.

In all 40 participants, the ratio of male and female was equal (20 male and 20 female), aged 22 ~ 34 years ( M=25.375 , SD=0.382 ). To ensure the retest reliability of the data, 30% of the sample was randomly selected to be retested one month later (Yuan et al., 2021). At the end of the study, each participant received ¥ 900 as a reward.

### 5.2 Materials

Referring Rueff-Lopes et al. (2015) and to Liu et al. (2022), the video that the participants had already labeled in Study 1 was selected as experimental material in Study 2. After discussion by the research team, the following criteria were adopted for the selection and Supplementary materials for the video. (1) Easy to understand: the videos were selected to be recorded within 3 ~ 5 mins as much as possible. The reason for this is that if the service delivery is too long, it is easy to cover multiple emotional states, making it difficult to evaluate a stable and unique emotional label for each data. (2) Representative: the selected video should have abundant obvious emotional fluctuations at different stages of the phone call (e.g., before and after the call), and can efficiently reflect the topic of the database. (3) Explicit: the selected video should show the delivery of the service in Mandarin, avoiding the use of dialect as much as possible.

#### 5.2.1 Video dataset

The emotion video dataset was constructed from the labeling outcomes of the 20 Ph.D. students in Study 1, and a 75% consistency level was the retention criterion (David et al., 2022; Liu et al., 2022). Meanwhile, the existence of emotional labor and the phenomenon of unconscious emotional expressions cannot be ignored (Hochschild, 1979; Rueff-Lopes et al., 2015; Sparks et al., 2015; Grandey and Melloy, 2017; Sun et al., 2018; Liu et al., 2022). Therefore, with the help of Adobe Premiere Pro, we intercepted and retained the additional video records from 0 ~ 15 s before- and after- the normal call, which were used to record the actual emotional expressions of the service staff. Eventually, 245 video files were obtained. Of these video files, 58 were between 0 ~ 1 min, 132 ranged from 1 ~ 3 mins, and the remaining 55 were over 3 mins.

#### 5.2.2 Audio dataset

The audio dataset was mainly downloaded from the Ocean Engine of the company and then matched with the video records. In practice, the post-sales staff usually provide secondary service to customers via personal mobile phones or landlines. Therefore, only 191 audio files were downloaded that could be matched to the video. In addition, because some of the background voices were too noisy and contained dialectal communication, we eliminated 23 and 29 recordings, respectively. In the end, 139 audio data were obtained. Of these audio files, 19 were between 0 and 1 min, 89 ranged from 1 to 3 min, and the remaining 31 were longer than 3 mins.

In summary, based on data matching, a total of 139 sets of emotional interaction data were obtained for this study. Among them, the duration of the video and audio was 5.47 and 5.18 h of playback, respectively.

### 5.3 Procedure

Pre-experiment. The indicators were explained to the participants to ensure that they understood the meaning of emotional valence (unhappy-happy) and arousal (inactivity-activity). Then, the participants were asked to watch/listen to the video/audio and evaluate the emotional state of the customers and service staff, respectively, during the service delivery. The purpose of this stage was to help the participants establish a stable rating scale. Finally, a threshold test was carried out. The differences between the participants’ evaluation results and the standard supported by the research team were compared. If the difference met the threshold requirement, the participant proceeded to the formal experiment, otherwise, this process was repeated until the requirement was met.

**Figure 5:**
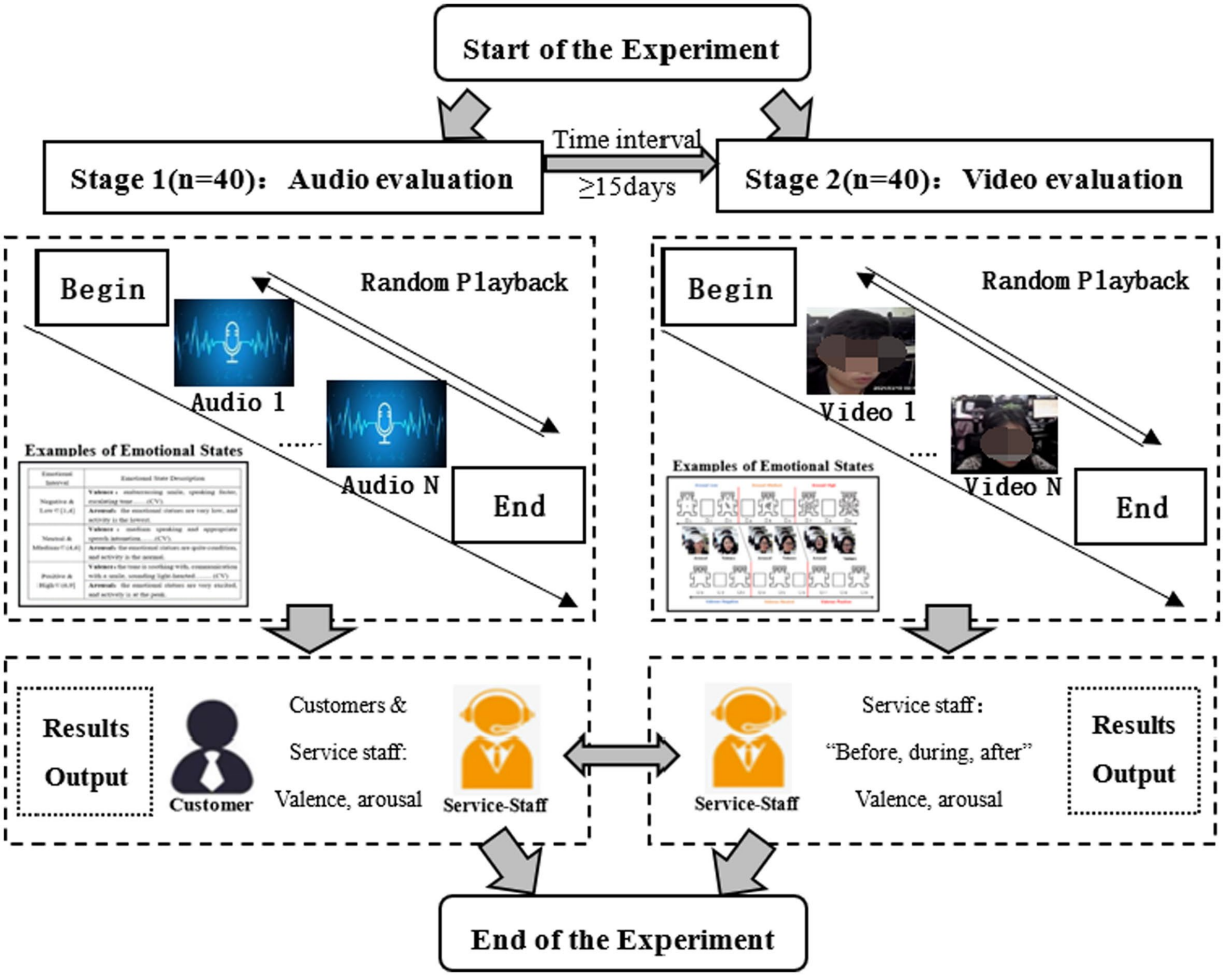
The flowchart of experimental.

Formal experiment. Participants were uniformly placed in a laboratory with identical lighting, no disturbances, and cleanliness. Before the experiment, the resolution of the computer monitor was adjusted to 1,920 × 1,080 by the members of the research team. In order to minimize the learning effect on the participants, the study was divided into two stages, separated by 15 days. Stage 1 was the evaluation of audio. Participants were asked to quickly evaluate the emotional state of customers and service staff during the service delivery. In Stage 2, the video’s evolution was completed. Participants were required to evaluate the emotional state of service staff during three phases, that is, before-, during-, and after- the call. Meanwhile, the text-based and picture-based scales constructed in Study 1 were provided to each participant as an auxiliary tool. Finally, to reduce the effect of fatigue, the study required the participants to take a break after a certain number of labels (see Figure 5).

Post-experiment. The outputs of audio and video were recorded (for recording rules see Table 1 and Figure 4). As for the degree of emotional interaction between customers and service staff, we defined the evaluation scores based on the before- and after- call as follows:

(1) If the scores of the emotional valence and arousal of service staff were always in the same numerical interval (e.g., 4 → 5, 7 → 8), we marked it as “Low.” (2) If any one of the indicators of emotional dimensions was in an adjacent numerical interval (e.g., 3 → 5, 5 → 7), we marked the emotional interaction as “Medium.” (3) If one of the indicators changed in a non-contiguous numerical interval (e.g., 1 → 7, 8 → 8), we marked the interaction as “High.”

### 5.4 Results

#### 5.4.1 Scoring results and validity tests

The Cronbach’s α of all 139 video and audio data was greater than 0.8, indicating a high level of consistency in participants’ scores. The results were statistically significant (see Table 2). Comparison of the emotional states of the service staff during the before- and after- stages of the call, 54 (38.85%) sets had a low level of emotional interaction, and 59 (42.45%) and 26 (18.71%) had a medium and high level, respectively. Thus, the CCSIAS database initially covers multiple degrees of emotional interaction states.

**Table 2:** Descriptive statistics for the emotional scores (*n* = 40).

Modalities	Emotional dimension	Calling stage/subjects	M ± SD	Min	Max	α
Video	Valence	Before-call	4.729 ± 0.855	2.750	7.500	0.941
		During-call	4.441 ± 0.856	2.273	6.909	0.938
		After-call	4.035 ± 1.197	1.917	7.667	0.961
	Arousal	Before-call	4.768 ± 0.828	2.750	7.000	0.917
		During-call	5.654 ± 0.578	4.545	7.273	0.844
		After-call	5.742 ± 1.082	3.166	8.333	0.953
Audio	Valence	Service staff’s state	4.861 ± 0.709	2.545	6.273	0.929
		Customers’ state	4.266 ± 0.833	2.091	6.001	0.936
	Arousal	Service staff’s state	5.799 ± 0.659	4.273	7.364	0.921
		Customers’ state	5.281 ± 1.381	2.012	7.909	0.974

On the 30th day after the end of the experiment, 12 (30%) participants were randomly selected to be retested. The correlation coefficient between the first and second test scores was r=0.881,P<0.001 , which meant that the reliability of the retest was adequate.

#### 5.4.2 Distribution of valence and arousal of CCSIAS

**Figure 6:**
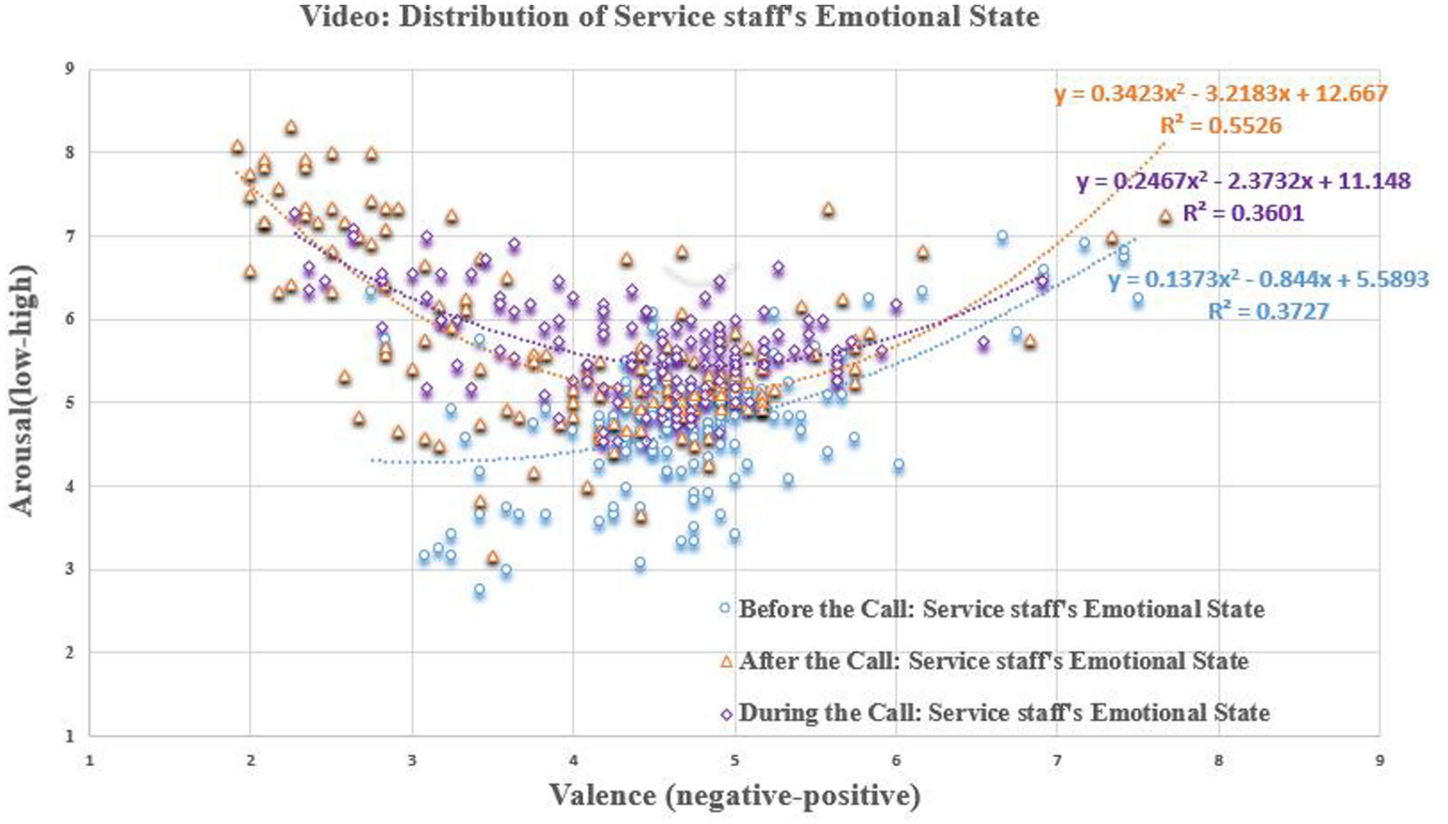
Scatter plot of scoring results (Video). Equation represents a quadratic regression fit of the data distribution.

In the video dataset (see Figure 6), three types of emotional information were combined: facial expressions, body actions, and communication voice (Liu et al., 2022). We found that the emotional valence ( $F=17.522,P<0.001,\eta^{2}=0.078$ ) and arousal ( $F=55.221,P<0.001,\eta^{2}=0.211$ ) of the service staff showed significant differences during the before-, during- and after-call stages. Specifically, the sample mean of service staff’s emotional valence before-call was ( M=4.729,SD=0.855 )>, during-call was ( M=4.441,SD=0.856 )>, after-call was ( M=4.035,SD=1.197 ), whereas emotional arousal showed before-call ( M=4.768,SD=0.828 )<, during-call was ( M=5.654,SD=0.578 )<, after-call was ( M=5.742,SD=1.082 ). The results show that, as the service continues, the emotional valence of service staff changes from positive to negative, while the arousal shows a change from low to high. In other words, the service delivery process has a significant influence on service staff’s emotions.

**Figure 7:**
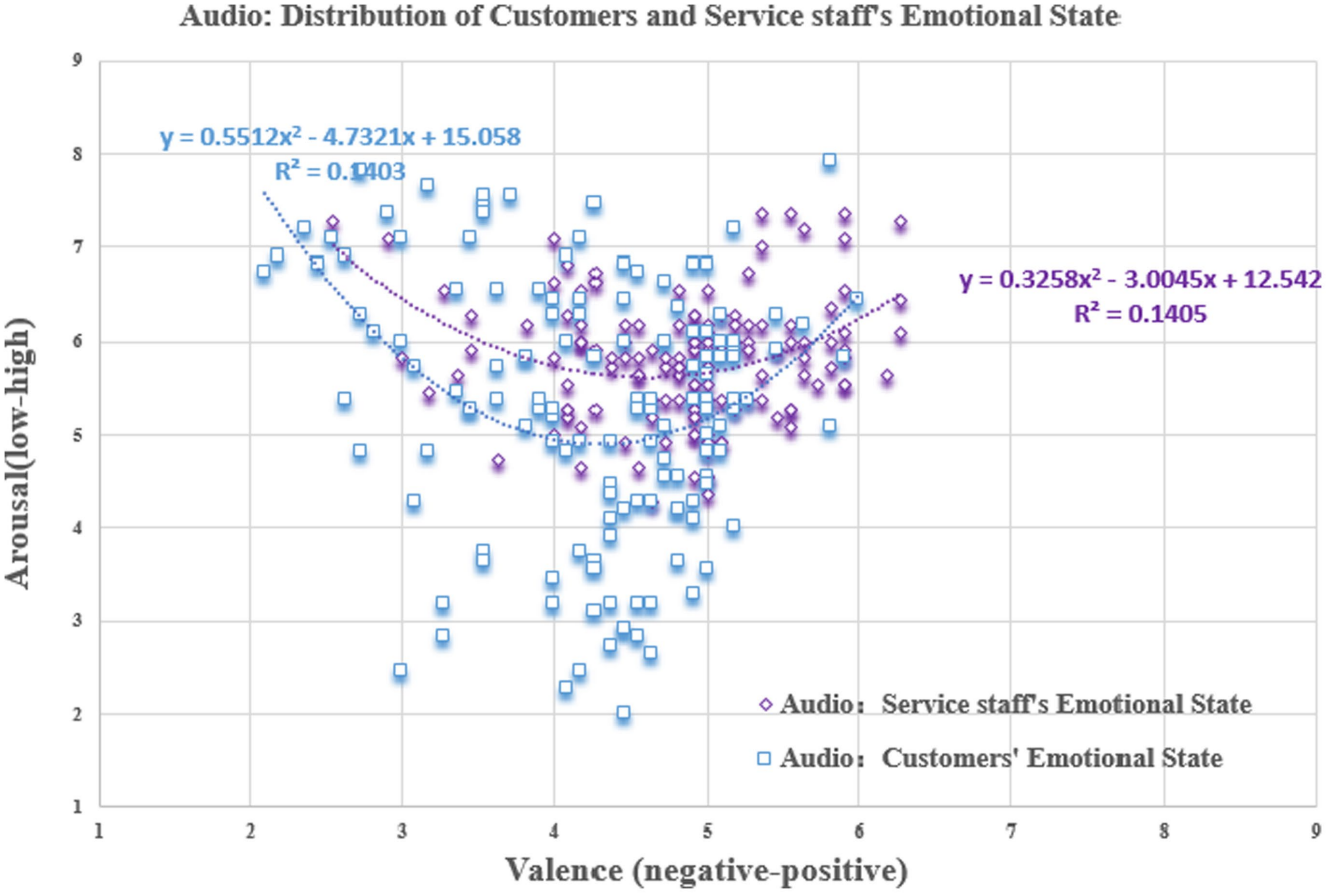
Scatter plot of scoring results (Audio). Equation represents a quadratic regression fit of the data distribution.

In the audio dataset (see Figure 7), the two types of emotional information, facial expressions, and body actions, were excluded. The emotional valence ( $F=41.054,P<0.001,\eta^{2}=0.129$ ) and arousal ( $F=15.997,P<0.001,\eta^{2}=0.234$ ) of the customers and service staff were significantly different. Specifically, in terms of the data dispersion, the emotional arousal of the two parties showed a trend for customers’ arousal ( SD=1.381 )to be > service-staff ( SD=0.659 ). However, in terms of the sample mean, the emotional valence of the service staff ( M=4.861,SD=0.709 ) was closer to neutral, while that of the customers ( M=4.266,SD=0.833 ) was significantly lower than that of the service staff. This result suggests that service staff not only consciously hide their true emotions during the actual service delivery, but are also negatively influenced by the customers. This is consistent with the findings of previous studies (Du et al., 2011; Rueff-Lopes et al., 2015; Rosalie et al., 2020; Liu et al., 2022).

We also compared the scoring results of the video and audio datasets in the during-call stage of service delivery (see Figures 6, 7). We found that the emotional valence ( $F=19.898,P<0.001,\eta^{2}=0.129$ ) of service staff showed a significant difference in the two datasets, while their arousal ( $F=3.846,P=0.051,\eta^{2}=0.014$ ) was on the borderline of significance. In the quantity of emotional information, this means that facial expressions and body actions are the main carriers of emotional valence in the service staff. However, due to the existence of emotional labor, it is impossible to identify the true emotions of service staff by relying on the communication voice. The findings also indicate that the study of emotional interaction is a multimodal issue, and that it is reasonable and necessary to construct a multimodal emotional database on visual and auditory channels in this study. Meanwhile, Table 2 shows that the score of each data has a moderate range of extreme differences, and basically covers the three emotional categories of negative & low, neutral & medium, positive & high, indicating that CCSIAS can be applied to activate and stimulate various emotions.

#### 5.4.3 Distribution of the degree of emotional interaction in CCSIAS

**Figure 8:**
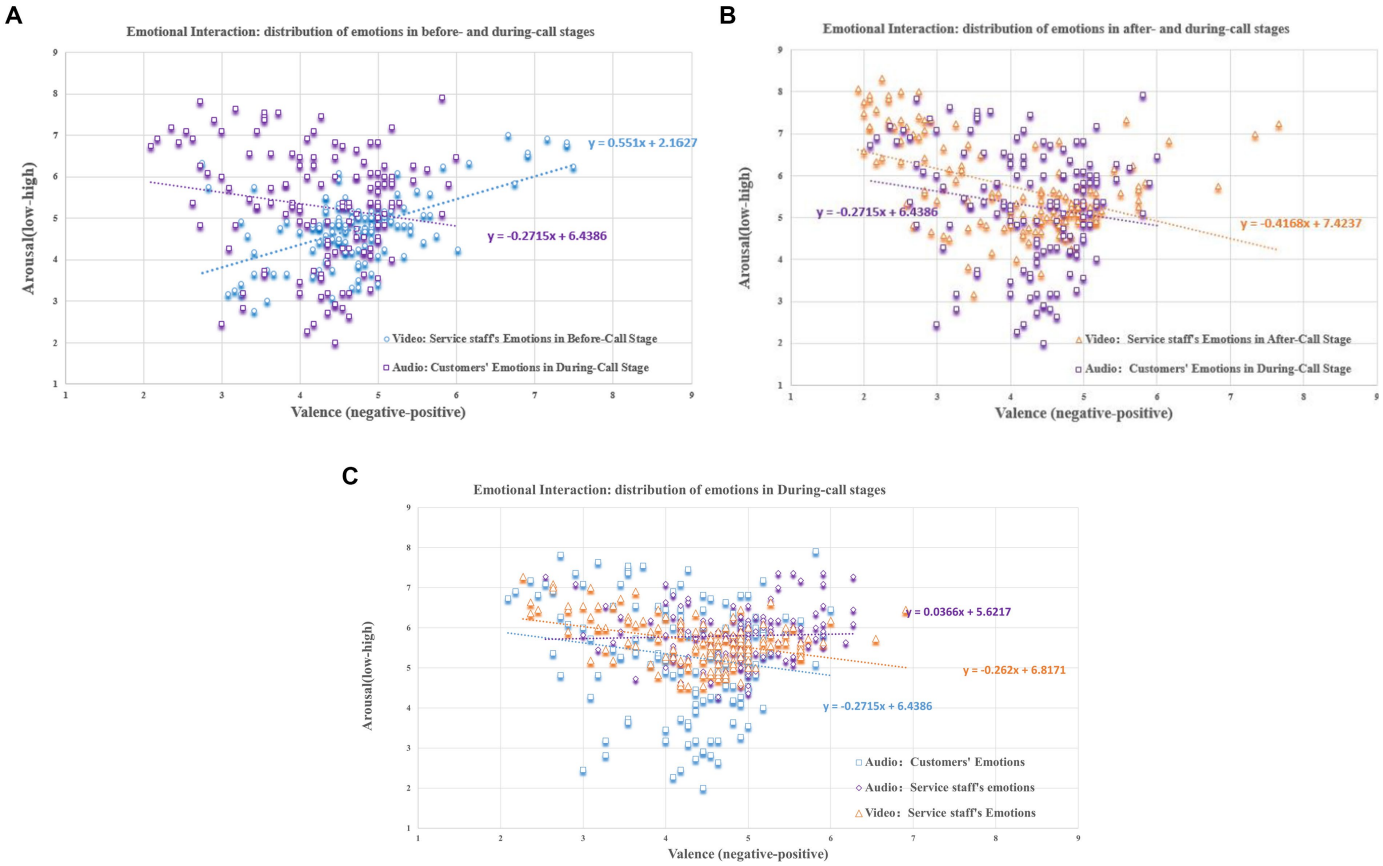
Emotional interaction between customers and service staff. **(A)** Before- and during-stages of service delivery. **(B)** During- and after-stages of service delivery. **(C)** During stage of service delivery.

By analyzing the ratings of the 139 datasets, it was found that the service staff emotions showed stages-specific characteristics in the three phone calls (see Figure 8). This indicates that the phenomenon of the emotional interaction is supported.

In the before- and during- stages of service delivery (Figure 8A). The emotional valence of service staff in the before-call stage was significantly higher than that of the customers in the during-call stage ( t=4.524,P<0.001 ). However, the emotional arousal of the service staff was significantly lower than that of the customers ( t=−3.893,P<0.001 ). In both stages, the interaction of the linear regression curves and the ANOVA results showed that the valence and arousal of the two parties were significantly different. This indicates that the emotional state of the service staff was not influenced by the customers before the service was provided, which is consistent with reality.

In the during- and after- stages of service delivery (Figure 8B), the emotional valence of the service staff in the after-call stage was lower than that of the customers in the during-call stage ( t=−2.295,P<0.05 ). However, the emotional arousal of the service staff was significantly higher than that of the customers ( t=3.165,P<0.01 ). The ANOVA results showed that there was no significant difference in the emotional valence ( $F=3.722,P=0.055,\eta^{2}=0.013$ ), while there was in emotional arousal ( $F=9.694,P<0.01,\eta^{2}=0.034$ ). This indicates that the customers’ emotional state plays a dominant role throughout the service process, and is mainly expressed through emotional arousal (Osgood, 1966; David et al., 2022). As time passes, especially within a few minutes after the end of the service delivery, service staff usually release their negative emotions by communicating and complaining with their colleagues (Rueff-Lopes et al., 2015; Sun et al., 2018). Furthermore, combining the heterogeneity of emotional information in the video and audio, as well as the slope of the linear regression curve, it can be easily inferred that facial expressions and body actions are the main channels through which the negative emotions are released by service staff in the workplace.

In the during-stage of service delivery (Figure 8C), the emotional valence ( t=1.998,P<0.05 ) and arousal ( t=3.063,P<0.01 ) of the service staff were both higher than that of the customers. Also, the valence ( $F=20.201,P<0.001,\eta^{2}=0.089$ ) and arousal ( $F=11.174,P<0.01,\eta^{2}=0.051$ ) of the two parties based on the video and audio were not significantly different, and the slopes of the linear regression curves remain largely consistent (orange and blue curves). However, the regression curves for the emotional state of service staff in the audio show an interaction effect with the regression curves for the customers (blue and purple curves). This suggests that service staff are susceptible to customers and show a consistent tendency in the service delivery. It also proves that multimodal emotional datasets are the best tool for scientific study of the problems in the phenomena of emotional interaction between customers and service staff.

## 6 Study3: a pilot test of CCSIAS in social participants

Study 3 aims to test the stability of the data labels in CCSIAS by recruiting volunteers to evaluate the 139 sets of data formed in Study 1 and Study 2.

### 6.1 Methods

#### 6.1.1 Participants

One hundred thirty four participants were openly recruited to engage in our experiment through the WeChat moments, Baidu Tieba, Sina-Weibo etc. Before the experiment, 9 participants withdrew for personal reasons. After the experiment, 7 participants’ data completion was missing or illegible, so a total of 16 participants were excluded. The final effective sample size was 118 (88.06%). Of these participants, 48 and 70 were male and female respectively, aged 18 ~ 55 years ( M=25.669,SD=0.778 ); 83.051% (=98÷118) had a university degree. All participants were non-psychiatric and had normal or corrected vision and hearing.

### 6.2 Materials

Study 3 used 139 sets of audio and video data evaluated by 40 MSc and PhD students in Study 2 as experimental material. The participants were of a wide age range and had various levels of understanding of the emotional indicators. Thus, to help the participants understand the meaning of the indicators more quickly and accurately, based on Osgood (1966)‘s definitions, the research team chose the Yellow Face Emoji and mammalian facial expressions (e.g., tiger) as tools for interpreting the emotional indicators (see Table 3).

**Table 3:** Participants’ auxiliary scoring scales (*n* = 118).

Dimension	Valence: unhappy← → happy			Arousal: inactive← → active		
Labels & Intervals	Negative	Neutral	Positive	Low	Medium	High
	[1,4]	(4,6]	(6,9]	[1,4]	(4,6]	(6,9]
Examples	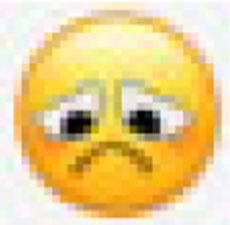	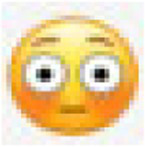	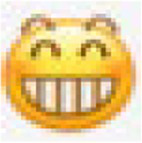	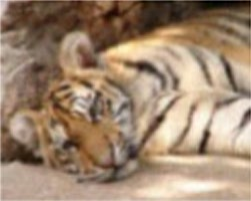	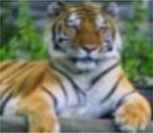	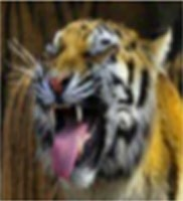 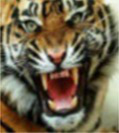

To check the validity of the emotional interaction labels and scores in Study 2, two parts of the prompts, emotional interaction and emotional recognition, were used to guide participants’ evaluation directions (Zhou et al., 2021). Specifically, for the audio, the participants were asked to answer two questions: (1) According to your feelings, what numerical interval do you think best matches the emotional state of the customers and service staff in this audio? (2) In your opinion, what is the intensity of this emotional state? For the video, the participants were asked to answer three questions: (1) According to your feelings, what numerical interval do you think best matches the emotional state of the customers and service staff in the three service stages (before-, during-, after-call) in this audio? (2) In your opinion, what is the intensity of this emotional state in the three stages? (3) In your opinion, is the difference in emotions of the service staff between before- and after-call stages mainly caused by the service delivery process? The intensity option is a 9-Likert scale, with 1 point indicating the lowest emotional intensity (very negative or low) and 9 points indicating the strongest emotional intensity (very positive or active).

### 6.3 Procedure

First, the experiments were conducted in both offline and online modes. Participants who were able to participate in the offline experiments were uniformly placed in a laboratory with identical lighting, no disturbances, and cleanliness (same as in Study 2). The online experiments were conducted via Tencent conferences, where members of the research team broadcast the materials online. Second, in order to minimize the common method bias, the experiments were uniformly played using QQ player, from 9:00 am to 12:00 am and 14:00 pm to 17:00 pm every day.

Furthermore, to avoid the influence of initial emotion on the evaluation, we randomly selected 30 neutral pictures from CAPS (Bai et al., 2005), IPAS (Lang et al., 1997) and Internet to be used as emotion regulation tools. The pictures were presented to the participants in the same sequence to adjust the participants emotions to a neutral & medium level. Then, 10% of data from 139 sets were selected for the threshold test materials. If the participants’ scores met the requirements, they proceeded to the next experimental stage; otherwise, this process was repeated until the requirement was met.

The experimental procedure was similar to Study 2 (see Figure 5). Participants were asked to evaluate two sets of data, audio and video, in two separate stages. At the end of the experiment, each participant received ¥150 as a reward.

### 6.4 Results

#### 6.4.1 Scoring results and data distribution

Table 4 shows that Cronbach’s α values of all 118 participant’s scoring results were greater than 0.8 and the intra-group correlation coefficient was great than 0.75. These data indicated that the scoring results of the two groups of participants (Study 2 and Study 3) had a good level of internal consistency (see Table 4).

**Table 4:** Descriptive statistics and the results of ANOVA (*n* = 118).

Modalities	Emotional dimension	Calling stage/subjects	M ± SD	α	ICC	*F*	sig
Video	Valence	Before-call	4.819 ± 0.731	0.963	0.929	0.881	0.349
		During-call	4.328 ± 0.615	0.967	0.936	1.554	0.214
		After-call	4.874 ± 0.622	0.852	0.845	1.471	0.266
	Arousal	Before-call	4.266 ± 1.035	0.846	0.824	0.213	0.645
		During-call	5.075 ± 0.499	0.923	0.857	0.285	0.594
		After-call	5.595 ± 0.942	0.967	0.936	1.459	0.228
Audio	Valence	Service staff’s state	5.168 ± 0.622	0.931	0.870	3.658	0.057
		Customers’ state	4.293 ± 0.752	0.943	0.892	0.828	0.364
	Arousal	Service staff’s state	5.738 ± 0.462	0.910	0.835	0.082	0.775
		Customers’ state	5.241 ± 1.301	0.962	0.927	0.061	0.804

**Figure 9:**
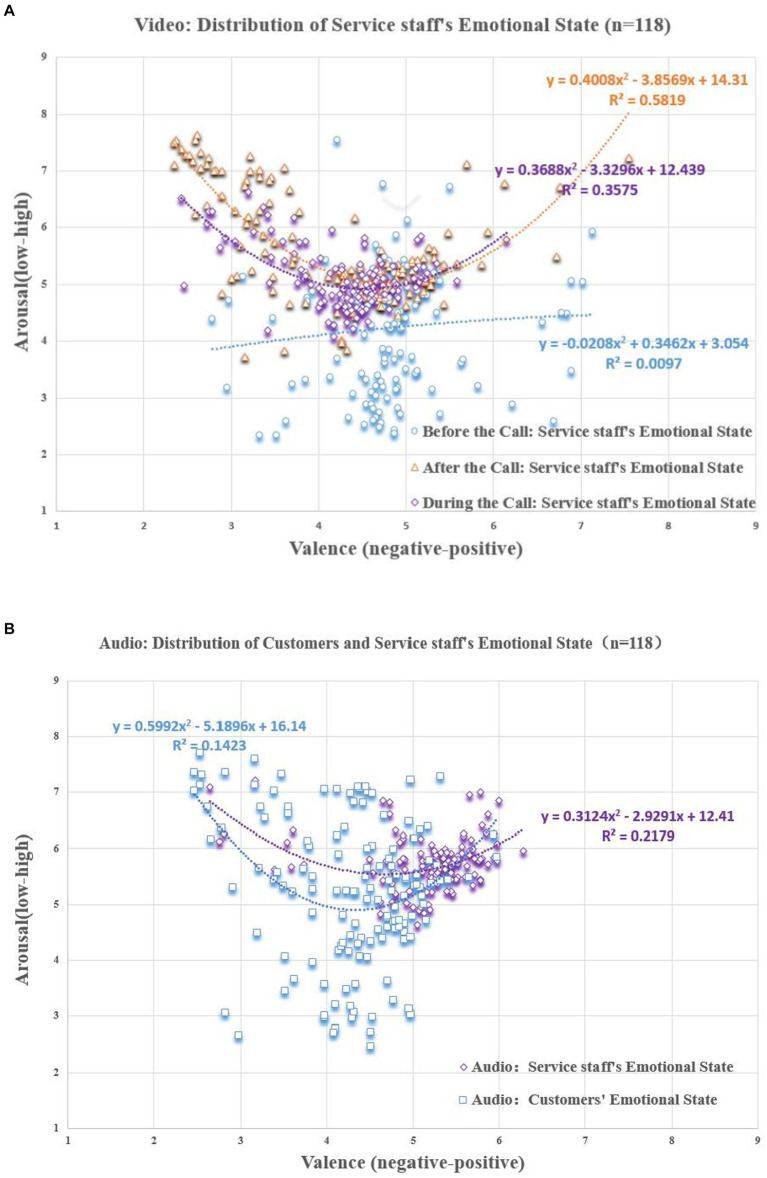
Distribution of scoring results of the 118 participants. **(A)** video dataset. **(B)** audio dataset.

Video dataset. In both scoring results (Figure 9A), the ones for the valence and arousal of service staff during the service delivery in the before-call ( $F_{\mathrm{valence}}=0.881,P=0.349;F_{\mathrm{arousal}}=0.213,P=0.645)$ , during-call ( $F_{\mathrm{valence}}=1.554,P=0.214;F_{\mathrm{arousal}}=0.285,P=0.594$ ), and after-call ( $F_{\mathrm{valence}}=1.471,P=0.266;F_{\mathrm{arousal}}=1.459,P=0.228$ ) were not significant between the 40 students and 118 participants. This suggests that the two groups of participants had highly consistent results for the labeling of the data in CCSIAS across the two studies (Study 1 and Study 2). Thus, this further supports that the video dataset in CCSIAS has good stability and can be used to activate different emotional states.

Audio dataset. The ANOVA results for two groups of participants for the emotions of customers and service staff were not significant (see Figure 9B), indicating that the audio dataset in the CCSIAS also had good stability. However, the paired sample t-tests showed that participants’ judgements of customers’ emotions were not significant ( $t_{\mathrm{valence}}=-1.002,P=0.318;t_{\mathrm{arousal}}=1.067,P=0.288$ ), while the opposite was shown for the service staff ( $t_{\mathrm{valence}}=-9.961,P<0.001;t_{\mathrm{arousal}}=2.261,P<0.05$ ). In addition, the sample mean showed strong significance for the results of customers ( $r_{\mathrm{valence}}=0.923,P<0.001;r_{\mathrm{arousal}}=0.948,P<0.001$ ) and service staff ( $r_{\mathrm{valence}}=0.858,P<0.001;r_{\mathrm{arousal}}=0.892,P<0.001$ ) in all three stages of service delivery. This implies that solely depending on the emotional information of communication voice enables one to deduce the emotions of the customers accurately, though there exists a certain level of imprecision when deducing the emotions of service staff.

In summary, emotional information is the basis for effective determination of emotional states. In actual emotional activation experiments, the video dataset is more effective in conveying customer service emotions than the audio dataset, which is similar to the findings of Xu et al. (2010) and Rosalie et al. (2020). Conversely, audio data is much more effective than video data when it comes to conveying service staff’s emotion. This is consistent with the data collection of CCSIAS, and again justifies that it is necessary to construct a multimodal database.

#### 6.4.2 The test of emotional interaction

To verify the stability of the labels of emotional interaction in the CCSIAS database, a t-test was conducted on the data from 40 MSc and PhD students and 118 participants at the before-call and after-call stages of the service delivery. The data showed that the valence ( $t_{\mathrm{before}-\mathrm{call}}=-3.386,P<0.01;t_{\mathrm{after}-\mathrm{call}}=-7.411,P<0.001$ ) and arousal ( $t_{\mathrm{before}-\mathrm{call}}=5.511,P<0.001;t_{\mathrm{after}-\mathrm{call}}=4.770,P<0.001$ ) of the service staff were significantly different in both groups. Therefore, there is evidence to support the emotional fluctuation of service staff during the before-call and after-call stages of service delivery.

At the same time, according to the inference in Study 2, the service staff’s emotional state in the during-call and after-call stages of the video dataset was set as the independent and dependent variable, respectively, and the customer’s emotional state in the audio data was set as the moderating variable. The moderating effect was then tested using the PROCESS plug-in to SPSS. The result showed that the interactive effect of service staff × customer was significant at the 95% confidence level, and the Sig F Chang value for the video and audio dataset was also significant ( $P_{\mathrm{video}-\mathrm{valence}}=0.032,P_{\mathrm{video}-\mathrm{arousal}}=0.014\text{;}$ $P_{\mathrm{audio}-\mathrm{valence}}=0.041;P_{\mathrm{video}-\mathrm{arsoual}}=0.045$ ). Therefore, there is evidence to support that the emotional state of service staff in the after-call stage in CCSIAS is primarily influenced by the emotional state of the customers in the during-call stage. Furthermore, the method used in this research to calculate the degree of emotional interaction is valid.

Based on the phenomenon of emotional interaction between customers and service staff in the service delivery, we set up wide-angle cameras to collect video data and downloaded audio data from the company’s Ocean Engine cloud database. Multimodal datasets (video-visual and auditory, audio-auditory) were used to construct a Chinese customers and service staff emotional interactive affective system (CCSIAS). To validate the degree of activation of emotions, Study 3 piloted CCSIAS with 118 participants. The results show that the CCSIAS has a high internal consistency of data labels, good retest reliability, and a wide distribution of different emotion categories. Therefore, the CCSIAS has reliability and broad applicability and could provide a database for future researchers studying multimodal emotional interaction issues.

## 7 Discussion

### 7.1 Final conclusions

This study developed a multimodal emotion database based on formal work scenarios, China Customer Service Interaction Emotional System (CCSIAS), and conducted preliminary analysis of the database through three studies.

In Study 1, this study generated text records to record the emotional state of service staff, and based on this, constructed text and image scales to evaluate the true emotional state of service staff.

In Study 2, we used the developed scales in Study 1 to rate the emotional status of service staff during the service process in the database, and found that the service staff emotions showed stages specific characteristics in the three phone calls. This indicates the existence of emotional interaction in the work process of service staff. Throughout the entire service process, the emotional state of customers plays a dominant role and can influence the emotions of service staff, so that as service time goes on, the emotions of service staff gradually show a trend consistent with those of customers.

In Study 3, we once again validated the emotional fluctuations of service staff. The results indicate that the CCSIAS has reliability and broad application, providing a database for future researchers studying multimodal emotional interaction issues.

### 7.2 Research contribution

The main contributions of this study are as follows:

First, this article enriches the literature on multimodal emotional interactions in the fields of organizational behavior and psychology. The existing literature on multimodal emotions has mainly focused on the development of computer deep learning algorithms and intelligent education research, such as extracting of contextual features to explain how virtual environments affect the learning behavior (Allcoat and Mühlenen, 2018), solving learning puzzles through emotional interactions (Huang et al., 2019), and eliciting deep emotional engagement (Hod and Katz, 2020) etc. Most researchers have focused on the effectiveness of developing algorithms for emotion recognition, but few studies have analyzed how the emotional interactions between customers and service staff affect each other’s psychology and behavior (Lahat et al., 2015; Liu et al., 2022). Therefore, based on the multimodal recognition method, this research is the first one to collect real emotion data in the workplace, and to construct a set of multimodal datasets (video-visual and auditory, audio-auditory). By comparing the scoring results of video and audio, we found that service staff not only consciously hide their real emotions during the service delivery, but also tend to be affected by the customers’ negative emotions. This is consistent with previous findings (Rueff-Lopes et al., 2015; Liu et al., 2022). Moreover, due to the existence of emotional labor (Hochschild, 1979), the customers’ emotional states dominate the whole process of service delivery. In order for service staff to alleviate their negative emotions, there are mainly two channels: facial expressions and body actions. Overall, this study greatly extends the study of emotional interaction topics by constructing a multimodal emotional dataset, which can provide multimodal data for subsequent studies.

Second, this research provides a reference for the development of a suitable tool for measuring video and audio emotion information. In the existing literature, the majority of the emotion measurement tools are text-based scales, such as PANAS, SDS, PAD etc. (Liu et al., 2022). While such tools provide a scientific basis for the effective assessment of emotional states, they often lack intuitive guidance when used to assess continuous, dynamic customer service emotional states in video data. Therefore, this article combines the SAM and two emotional dimensions (Bradley and Lang, 1994; Lang et al., 1997), valence and arousal, and real emotional pictures of service staff in the workplace, to develop a set of picture-based and text-based scales for evaluating the emotional state between two parities (see Table 1 and Figure 4). This study enriches the perspective of emotion assessment tools, and subsequent research can be built on this idea to develop emotion assessment tools for other domains and scenarios.

Third, this study proposes and validates that the study of the phenomena of emotional interaction between customers and service staff is a multimodal issue. Although the literature has indicated that the research of emotional issues should be based on a multimodal perspective (Kress, 2010; David et al., 2022), few scholars have conducted multimodal analyses of emotional interaction phenomena. In this article, we found that a video dataset is significantly better than an audio one in conveying the emotional information of service staff (see Figure 9). The video dataset, which included three types of emotional information, facial expressions, body actions, and communication voice, was better at activating participants to perceive the emotional state related to service staff than the audio dataset. This suggests that the traditional method of assessing the emotional affective system based on single-sensory data (e.g., picture-visual, music-auditory, text-visual) is limited. Improving the efficiency of emotional activation must be achieved by relying on multimodal databases. Therefore, the CCSIAS built in this article can provide better data support for future research in organizational behavior, psychology, and even the development of computer deep learning algorithms.

Finally, we found that the real emotion states of the service staff tended to change from “arousal-low, valence-positive” to “arousal-high, valence-negative” as the service time changed, whereas the customer’s emotions tended to be “arousal-low, valence-negative” or “arousal-high, valence-negative” throughout the service delivery process. The customers’ emotions were highly dominant and negatively influenced the service staff over time, which was consistent with previous research findings (Rueff-Lopes et al., 2015; Sun et al., 2018). Overall, this study has constructed a CCSIAS based on a real workplace that can provide literature and data support in theoretical and practical areas focusing on emotional interaction issues.

### 7.3 Limitations and future directions

Of course, of this study has limitations, and future research may need to focus on the following points:

First, Study 1 constructed a set of text-based scales and picture-based scales, of which the text-based scales were based on high-frequency word statistics. This type of statistic is a general way of describing the emotional state of service staff. However, the expression of emotions varies from person to person. Therefore, the text-based and picture-based scales need to be revised and adapted in practice.

Second, 40 MSc and PhD students were recruited to compile the database in Study 2, in order to maximize the stability and scientific validity of the emotion labels. However, due to the complexity of emotions, the time duration of the service delivery, the actual difficulty of scoring, and the financial constraints of the research team, only 139 sets (278 in total) of stable data were developed based on 15 consecutive days of data. The amount of data is a relatively small. In addition, there was no access to video data of the customers during the call, so the CCSIAS results on the “emotional interaction level” were based only on the difference in the participants’ scoring of the emotional state of the service staff during the before- and after-call stages of service delivery. Therefore, this study did not investigate how the degree of emotional interaction is influenced by the customers and what the underlying mechanism model is. Future research can provide answers to these questions.

Third, it was shown in Study 3 that the emotional phenomena of customer service were mainly caused by the customers and that the sample mean and moderating effects showed significance. However, social influence theory and emotional event theory (Rupp and Spencer, 2006; Luo et al., 2019)suggest that the service delivery behavior of online customer service occurs in the offline workplace. So, it remains unknown how other elements in the workplace (e.g., communication between colleagues, tattling, etc.) moderate the real emotions of service staff. Future research could explore this in more depth.

## Data availability statement

The original contributions presented in the study are included in the article/Supplementary material, further inquiries can be directed to the corresponding author.

## Ethics statement

Written informed consent was obtained from the individual(s) for the publication of any potentially identifiable images or data included in this article.

## Author contributions

PL: Writing – original draft, Conceptualization. YZ: Writing – original draft. ZX: Writing – review & editing. YG: Writing – review & editing.
